# Increases in solar conversion efficiencies of the ZrO_2 _nanofiber-doped TiO_2 _photoelectrode for dye-sensitized solar cells

**DOI:** 10.1186/1556-276X-7-98

**Published:** 2012-02-02

**Authors:** Jiao Wang, En Mei Jin, Ju-Young Park, Wan Lin Wang, Xing Guan Zhao, Hal-Bon Gu

**Affiliations:** 1 Department of Electrical Engineering, Chonnam National University, Gwangju, 500-757, South Korea; 2 Southwestern Research Institute of Green Energy Technology, Mokpo-si, Jeonllanam-do, 530-400, South Korea

**Keywords:** zirconia nanofiber, titania, DSSC

## Abstract

In this paper, in order to improve the efficiency of dye-sensitized solar cells, we introduced zirconia [ZrO_2_] nanofibers into a mesoporous titania [TiO_2_] photoelectrode. The photoelectrode consists of a few weight percent of ZrO_2 _nanofibers and a mesoporous TiO_2 _powder. The mixed ZrO_2 _nanofibers and the mesoporous TiO_2 _powder possessed a larger surface area than the corresponding mesoporous TiO_2 _powder. The optimum ratio of the ZrO_2 _nanofiber was 5 wt.%. The 5 wt.% ZrO_2_-mixed device could get a short-circuit photocurrent density of 15.9 mA/cm^2^, an open-circuit photovoltage of 0.69 V, a fill factor of 0.60, and a light-to-electricity conversion efficiency of 6.5% under irradiation of AM 1.5 (100 mW/cm^2^).

## Introduction

Dye-sensitized solar cells [DSSCs] have generated a considerable research interest because of their high-energy conversion efficiency (approximately 11%) and low production costs [1-3]. A typical DSSC device contains a light-harvesting layer on a photoelectrode and a Pt-coated layer on a counter electrode; both electrodes are made of a transparent conducting oxide substrate; an iodine-based electrolyte fills the space between the photoelectrode and the counter electrode to serve as a redox mediator in a sandwich-type structure. Performance of the DSSC depends on many factors such as the TiO_2 _surface morphology, particle size, thickness of the photoelectrode, nature of the dye, etc. [4-10].

A high light-to-electricity conversion efficiency results from a large surface area of the mesoporous TiO_2 _photoelectrode, on which the dyes can be sufficiently adsorbed. In this study, we introduced zirconia [ZrO_2_] nanofibers into the mesoporous titania [TiO_2_] photoelectrode. The ZrO_2 _nanofibers are prepared by electrospinning. The TiO_2 _film composite with ZrO_2 _nanofibers creates a larger surface area than the single TiO_2 _film, in which case the amount of dye loading was increased and short-circuit photocurrent density and solar conversion efficiency are also increased.

## Experimental details

**Figure 1:**
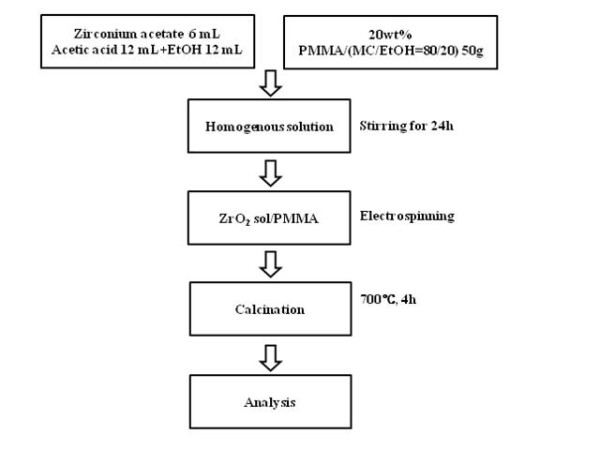
**Preparation of the ZrO_2 _nanofiber**.

The ZrO_2 _nanofiber additives were prepared by electrospinning method. At first, mixed together, 6 ml zirconium acetate, 12 ml acetic acid, 12 ml ethanol, and 50 g poly(methyl methacrylate) were stirred for 24 h; then, the compounds were sintered at 700°C for 4 h. A detailed process is displayed in Figure 1.

The TiO_2 _paste was prepared by mixing TiO_2 _with Degussa P-25, polyethylene glycol, acetyl acetone, distilled water, triton X-100, HNO_3_, and ZrO_2 _nanofibers. The concentrations of ZrO_2 _nanofibers were 0, 3, 5, and 7 wt.%. The mixed solutions were ball milled at 100 rpm for 10 h. The photoelectrode was fabricated using a clean fluorine-doped tin dioxide [FTO] (approximately 8 Ω/cm^2^, Pilkington conductive glass, Seoul, South Korea) by squeeze printing. The coated photoelectrode was heat treated at 450°C for 30 min with a heating rate of 5°C/min. The obtained photoelectrode was immersed into the ethanol solution containing [*cis*-diisothiocyanato-bis(2,2'-bipyridyl-4,4'-dicarboxylato)ruthenium(II) bis(tetrabutylammonium)] (N719 dye, Solaronix, Aubonne, Switzerland) for 24 h. The active area of the photoelectrode was 0.5 × 0.5 cm^2^. On the other hand, the counter electrode was prepared similar to the photoelectrode preparation. Pt-Sol (Pt catalyst/SP, Solaronix) was coated onto the FTO glass by the squeeze printing method. The coated paste was heat treated at 450°C for 30 min with a heating rate of 5°C/min.

The electrolyte solution consisted of 0.3 M 1,2-dimethyl-3-propylimidazolium iodide, 0.5 M Li(I), 0.05 M I_2_, and 0.5 M 4-t-butylpyridine in 3-methoxypropionitrile between the two electrodes. The dye-coated photoelectrode and the Pt-coated counter electrode were sandwiched using a 60-μm-thick hot-melt sealing foil (SX 1170-60, Solaronix).

The field-emission scanning electron microscope [FE-SEM] (S-4700, Hitachi, Seoul, South Korea) and BET were used to examine the morphology and the pore distribution volume of the TiO_2 _film. In order to investigate the physical and optical characteristics of the dye-adsorbed TiO_2 _films, the UV-visible [UV-Vis] spectrum measurement was performed. The photovoltaic properties were investigated by measuring the photocurrent-voltage characteristics under illumination with air mass [AM] 1.5 (100 mW/cm^2^) simulated sunlight.

## Results and discussion

**Figure 2:**
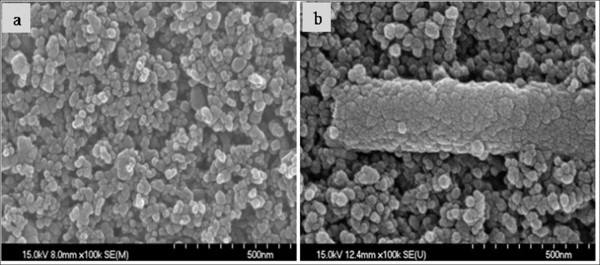
**FE-SEM images of (a) TiO_2 _film and (b) 5 wt.% ZrO_2 _nanofiber-doped TiO_2 _film**.

Figure 2 shows the FE-SEM images of the TiO_2 _film's surface and the 5 wt.% ZrO_2 _nanofiber-added TiO_2 _film's surface. In the ZrO_2 _nanofiber-added TiO_2 _film in Figure 2b, the ZrO_2 _nanofiber was shown at the surface of the TiO_2 _film, or the TiO_2 _film was studded with the ZrO_2 _nanofiber. The TiO_2 _film's surface area was increased, and the dye adsorption contents became larger by the addition of the ZrO_2 _nanofiber. So, we can forecast that the TiO_2 _electrode is able to obtain high conversion efficiency.

**Figure 3:**
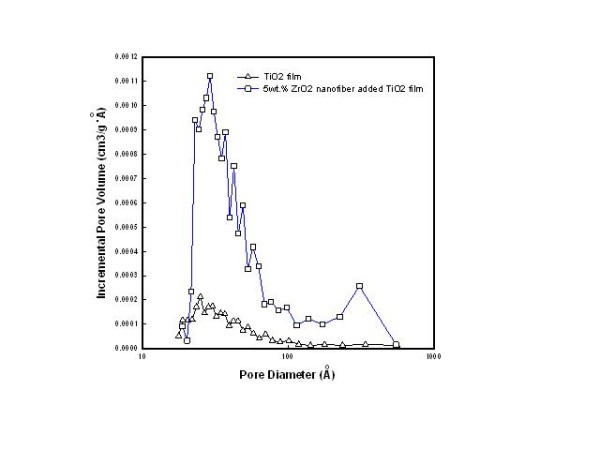
**BJH curve of TiO_2 _film (triangle) and 5 wt.% ZrO_2 _nanofiber-doped TiO2 film (square)**.

Figure 3 shows the pore distributions calculated from adsorption data using the Barrett-Joyner-Halenda [BJH] method. As shown in Figure 3, a broad peak was found at around 25 nm, and an added 5 wt.% ZrO_2 _nanofiber at around 30 nm was observed. Compared to the pure TiO_2 _film, the 5 wt.% ZrO_2 _nanofiber-added TiO_2 _films show a significant change in the pore size distribution. A large pore volume in BJH was observed on the TiO_2 _film with 5 wt.% ZrO_2 _nanofibers, which is in agreement with the results of the FE-SEM image.

**Figure 4:**
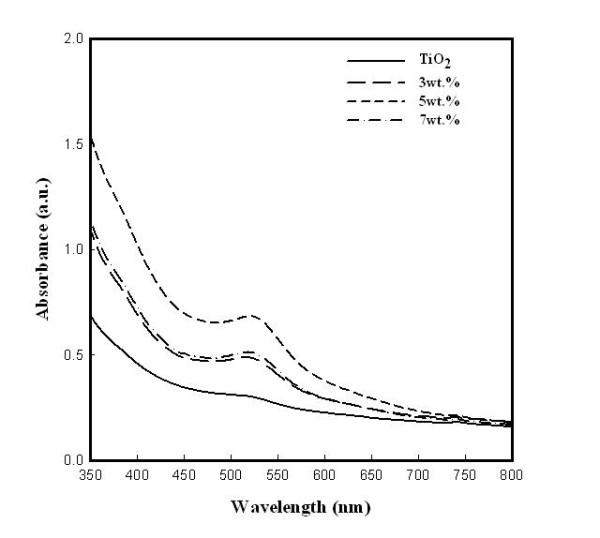
**UV-Vis spectrum of TiO_2 _film and ZrO_2 _nanofiber-doped TiO_2 _film after 24-h dye absorption**.

Figure 4 shows the UV-Vis absorption spectra of the dye-adsorbed TiO_2 _film and the 3, 5, and 7 wt.% ZrO_2 _nanofiber-doped TiO_2 _films. From the results, the absorption spectra increased at around 538 nm with added ZrO_2 _nanofibers doped in the TiO_2 _film and also enhanced the amount of dye loading. So, the 5 wt.% ZrO_2 _nanofiber-doped TiO_2 _film had the best dye loading, and also, its solar conversion efficiency was the best among the samples.

**Figure 5:**
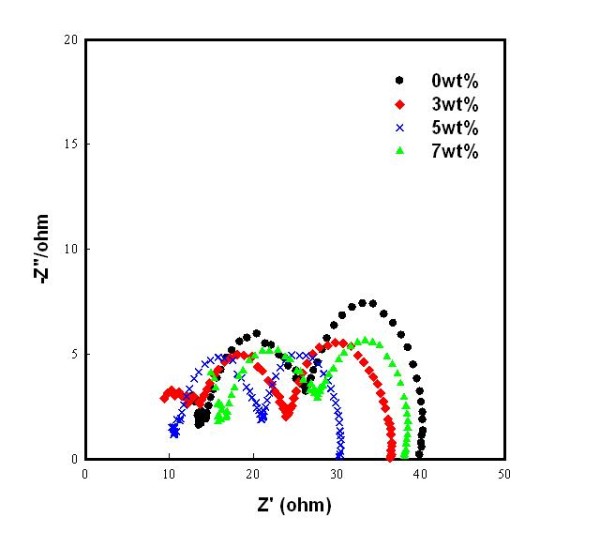
**Nyquist plots of the DSSCs using TiO_2 _film and ZrO_2 _nanofiber-doped TiO_2 _film**. Black circle, 0 wt.%; red diamond, 3 wt.%; cross mark, 5 wt.%; green triangle, 7 wt.%.

In order to determine the factors that influence the stability of the DSSCs, electrochemical impedance spectroscopy [EIS] was performed. Figure 5 shows that the Nyquist plot of EIS of the DSSCs exhibits semicircles, which are assigned to the electrochemical reaction at the Pt counter electrode, the charge transfer at the TiO_2_/dye/electrolyte interface, and the Warburg diffusion process of I^-^/I_3_^- ^[9,10]. As shown in Figure 5, the second semicircle is the resistance (*R*_2_) related to the electron transport in the TiO_2_/dye/electrolyte interface which is reduced. It can be seen that the TiO_2 _film and the 3, 5, and 7 wt.% ZrO_2 _nanofiber-doped TiO_2 _films are 13.2, 10.3, 9.6, and 11.9 Ω, respectively.

**Figure 6:**
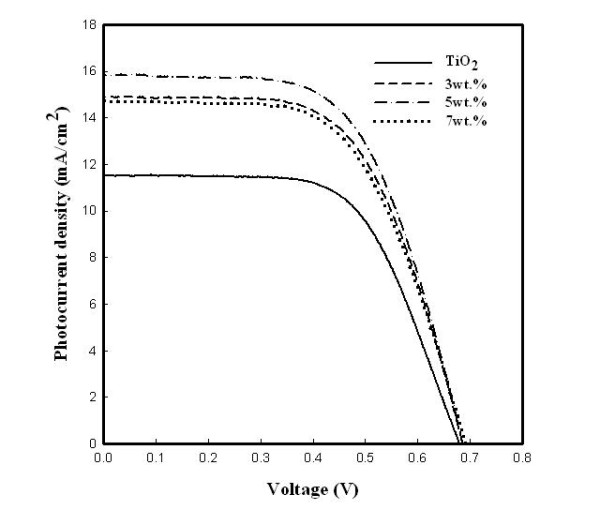
**Photocurrent-voltage curve of the TiO_2 _film and the TiO_2 _films with ZrO_2 _nanofibers**.

Figure 6 shows photocurrent-voltage characteristics of the DSSCs with the ZrO_2 _nanofiber-doped TiO_2 _film. The open-circuit photovoltage was almost the same, and the short-circuit photocurrent density increased with the added amount of ZrO_2 _nanofibers and had reached the maximum at 5 wt.% of ZrO_2 _nanofiber (15.9 mA/cm^2^). The open-circuit photovoltage [*V*_oc_], the short-circuit photocurrent density [*J*_sc_], the fill factor [FF], and the light-to-electricity conversion efficiency [*η*] at 5 wt.% added ZrO_2 _nanofiber were 0.69 V, 15.9 mA/cm^2^, 0.60, and 6.5%, respectively, as shown in Table 1. From the results, we can realize that the insertion of ZrO_2 _nanofibers creates a larger surface area and reduces the resistance of the photoelectrode, especially for the optimal amount of ZrO_2 _contents (7 wt.%) of the photoelectrode in DSSCs.

**Table 1:** Photocurrent-voltage characteristics of DSSCs using TiO_2 _with different amounts of ZrO_2 _nanofibers

Sample	*V*_oc _(V)	*J*_sc _(mA/cm^2^)	FF	*η *(%)
Pure TiO_2_	0.68	11.5	0.62	4.9
3 wt.% ZrO_2 _nanofiber-doped TiO_2_	0.69	14.9	0.60	6.2
5 wt.% ZrO_2 _nanofiber-doped TiO_2_	0.69	15.9	0.60	6.5
7 wt.% ZrO_2 _nanofiber-doped TiO_2_	0.69	14.7	0.59	6.0

*V*_oc_, open-circuit photovoltage; *J*_sc_, short-circuit photocurrent density; FF, fill factor; *η*, light-to-electricity conversion efficiency; TiO_2_, titania; ZrO_2_, zirconia.

## Conclusions

In summary, a ZrO_2 _nanofiber-doped TiO_2 _film was used as a photoelectrode in DSSCs. The ZrO_2 _nanofiber-doped TiO_2 _films had a larger surface area than the pure TiO_2 _film, in which case the amount of dye loading was increased, and *J*_sc _and *η *were also increased. The optimum ratio of the ZrO_2 _nanofiber was 5 wt.%. The DSSC with the 5 wt.% ZrO_2 _nanofiber photoelectrode provided the highest *η *of 6.5%, *J*_sc _of 15.9 mA/cm^2^, *V*_oc _of 0.69 V, and FF of 0.60 under AM 1.5 (100 mW/cm^2^) simulated sunlight illumination. Therefore, ZrO_2 _fibers are a promising additive for the realization of high-efficiency DSSCs.

## Competing interests

The authors declare that they have no competing interests.

## Authors' contributions

JW fabricated the DSSCs and UV-Vis analysis. EMJ was the paper chaser and performed the analysis of photocurrent-voltage characteristics and impedance. WLW performed the BET analysis. J-YP prepared the ZrO_2 _nanofibers. XGZ performed the FE-SEM analysis. H-BG was thesis director. All authors read and approved the final manuscript.
